# The Double-Aspect of Life

**DOI:** 10.3390/biology7020028

**Published:** 2018-05-07

**Authors:** Daniel Fels

**Affiliations:** Department of Environmental Sciences, University of Basel, Hebelstrasse 1, 4056 Basel, Switzerland; daniel.fels@hispeed.ch; Tel.: +41-61-322-1675

**Keywords:** electrostatic fields, electrodynamic fields, bioelectricity, ecology, evolution

## Abstract

Life is based on two aspects: matter and a non-material, electrical component. In a dynamic system of reciprocal causality, matter and the so-called bioelectricity interact with one another, forming a functional unity. The aim of this essay is to summarize evidence for bioelectricity, for the sensitivity of biosystems to external physical factors and for the interactions of internal bioelectricity with internal biochemical structures. I propose non-material information of bioelectrical states to be just as inheritable from generation to generation as is the material genetic code.

## 1. Introduction

Life expresses itself as a manifold of interactions between genes and phenotypes such as physiology, biochemistry, anatomy, morphology, life-history traits and behavior. Some of its features are visible or material, like the color patterns of eggshells or the number of eggs in a clutch; others are invisible, such as structural changes of enzymes, behavior or cognitive abilities. In any case, we attribute these features of life to the *material* organization of an organism. However, this material structure and its dynamic interrelationship with itself and the environment constitutes only one aspect of life. There is yet a second aspect that operates during an individual’s life and across generations: the bioelectric state of the organism [1].

Bioelectricity is generated by electrical charges of ions and molecules in an aqueous environment. Ions and oscillations of charged molecules as well as chemical reactions lead to electrical currents and electrostatic and electrodynamic fields. In the following, the term “electromagnetic fields” (EMFs) is used to refer to the last two types of fields mentioned above (note that studies on cell-internal DC currents are not the subject of this essay).

**Figure 1:**
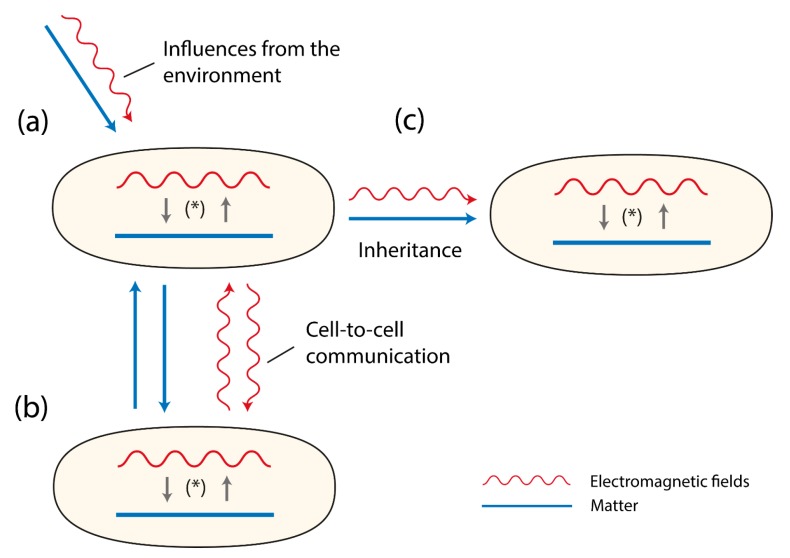
Three cells (organisms) are shown. They contain matter (depicted as a line) and EMFs (depicted as a wave-line). Both matter and EMFs interact with each other as a system of reciprocal causality, together building the evolving double-aspect of life (*). In (**a**) the sensitivity to environmental influences are presented depicting two types of influences, material ones (e.g., food, ions, parasites) and EMFs (e.g., cosmic rays, photons, IR-waves). In (**b**) the figures refer to the signaling between cells or organisms either via material components (e.g., pheromones, neurotransmitters, hormones) or EM-fields (e.g., photons). In (**c**) the figure displays that both matter and EMFs are inherited.

EMFs play essential roles in cell dynamics and are associated with the material aspect of the cell as is the gravitational field associated with mass [1]. The existence of cell-internal EMFs suggests physical constraints, including (i) the (above-mentioned) unavoidable generation of these fields, (ii) the impossibility of charged cell components to remain inert to these fields, and (iii) consequently, the looping-back of the fields on cell components leading to a system of reciprocal causality [2,3]. This interaction between the material components of the cell and the fields of the cell is proposed here as a system of reciprocal causality between the EMFs of the cell and the matter of the cell. Therefore, this essay speaks of the double-aspect of life (Figure 1).

The aim of this essay is to create awareness on the significant contribution of cell-internal EMFs on living processes. This will support the integration of cell-internal EMFs into an updated and extended theory of life.

## 2. The Existence of Cell-Internal Electromagnetic and Electrostatic Fields

Among many other great discoveries, the 19th century was characterized by the discovery and description of organic molecules, the so-called molecules of life (DNA, hormones, etc.). In the early 20th century, inspired by Einstein’s development of the field concept, the Russian morphologist A.G. Gurwitsch began looking for *field-transmitted effects* on development and growth (in addition to effects transmitted by molecules). He became famous for his discovery of what he described as *mitogenetic radiation*, referring to the induction of cell divisions from one plant root to the next across quartz-barriers. Using (normal) glass as a barrier, he found no effects. As normal glass does not transmit electromagnetic waves shorter than 350 nm, but quartz does, he concluded that a signal in the UV-range [4,5]. Yet, with these experiments, the ‘barrier-method’ was born, allowing physical but not chemical signals to be transmitted. Even though these experiments, as well as their results, had disappeared from mainstream biology over the years, they have recently been experiencing a renaissance [6].

When referring to such experiments today, we speak about photon-induced effects, i.e., of electromagnetic waves in the visible range inducing reactions in neighboring cell populations by which they are separated through physical barriers. There is knowledge accumulating about (i) the source of the signals, (ii) their effects, and (iii) the signal-transduction pathway from reception to eventual gene activation.

Reactions that are induced by *reactive oxygen species* (so called ROS) are considered to be the major source for endogenous photon-generation in living cells. Depending on the substrate, these reactions lead to different products that are associated with the energy-difference between substrate and product, namely EMFs of different frequencies (ranging from 400 to 700 nm, the THz region) [7,8,9]. Not surprisingly, it is in this context that increasing numbers of photoreceptors in cells have been reported [10,11,12], with evidence for molecular cascades from photoreceptors to gene activation [13], indirectly shown, for example, by measuring the protein increase in a neighboring cell population [14,15,16]. Note that effects were found for quartz-separation [15,16], as well as for glass-separation [14]. We conclude that the EMFs radiated by cells can function as signals, inducing reactions that belong to the organization of life.

Yet, a technique for studying all aspects of electromagnetic communication from the production of EMF-signals to their perception, and via molecular cascades to a final function, in an all-in-one system is still awaiting our cooperative efforts. However, already today we can distinguish functions induced by non-chemical signals of a most probably electromagnetic nature. Across chemical barriers that allow optical communication—e.g., EMFs—to be transmitted, we observed manifold functions, including the induction of cell growth across quartz-separators [17], inhibition of cell growth across quartz-separators, as well as induction across glass-separators [18], and recently, the endogenous physical regulation of cell density (no difference found between glass or quartz separation) [19]. Further, energy uptake is also under non-chemical influence (reduced energy uptake when quartz-separated, increased uptake when glass-separated) [18]. Not every non-chemical interaction must be due to fields; e.g., movement in an aqueous system has been shown to follow hydrodynamic interaction [20]. Yet, cell positioning on the two sides of the same thin glass plate was assumed to occur due to electromagnetic radiation [21], as was interpreted also for the induction of protein synthesis (see above) [14,15,16]. Evidence for directional growth across glass barriers was found for algal zygotes [22]. Moreover, effects have been found from one species to another, as well. These include growth (with quartz and glass separation) [23], as well as cell morphology with induction across Petri-dishes [24]. A theoretical concept capturing these aspects is the so-called cavity resonance; cell internal EMFs not only oscillate within cells, but also within a tissue, possibly leading to pattern formation, e.g., plant meristem development [25] (Figure 1b).

The list of effects from electric charges or oscillations affecting cells is not at its end, however. A remarkable example for electrostatic ecology can be seen in electrostatic pollination [26,27] (Figure 1a). Yet, we find electrostatic phenomena at a much smaller scale in life, as well. When a sperm encounters the ovum, successful fertilization only occurs after what is called a zinc spark. Only after an enormous membrane voltage change associated with this zinc spark event occurs, can embryonic development start successfully [28]. This membrane voltage dependency for life processes to occur continues in the development of multicellular organisms, and is found as a trigger for gene activation and epigenetic control [29], as well as for regeneration [30] or stem cell differentiation [31]; note that these studies are about (changing) resting potentials across cell membranes or animal bodies.

Membrane potentials are also responsible for the fact that our bodies are electrically charged [32] from the very onset of embryonic development. The long-standing question as to what is giving form to a body [33] is partly answered by this electric state of the embryo [34]; the electrical potential field between its poles is functioning as a guideline for migrating embryonic cells, which themselves are polar. They thereby bring their own charge to new positions in the embryo, thus changing locally *the shape* of the electric field of the embryo, which then might, with slightly altered *field-lines*, affect further electric paths (of course, this does not exclude chemically induced contact between cells) [34]. We note that A.G. Gurwitsch already suggested in 1912 a “Kraftfeld”, i.e., a field of force (author’s transl.) as a *conditio sine qua non* for cell guidance during embryo development [35].

While the above examples are rather unfamiliar, we know that membrane potentials along axons change in low frequencies, whereby millions of cells go into synchrony with each other producing typical brainwaves [36] (Figure 1). 

## 3. Effects of External Electromagnetic Fields on the State of Biosystems

The notion that cells are sensing external fields is already clear from the above-mentioned examples of EM-signals, polar embryos or brainwave synchronicity. It is therefore a small step to think of bioeffects that are induced by non-biological sources, i.e., a technical apparatus. This to either test the hypothesis (of non-chemical induction) or to use it for non-invasive therapeutic applications [37,38]. Note that some effects are already obtained when using very low frequency EMFs, e.g., of 3–30 Hz [37].

In fact, successful treatment with external EMFs is possible. This includes, e.g., wound healing and osteogenic differentiation, as well as bone growth and regeneration [39]. In this context, one must mention the concern coming from this knowledge, as we are surrounded by many different EMFs, some of which are detrimental to our health. … *“One can envisage that EMF “speaks” to each organism and each cell with a different language. The answer to that call can potentially induce protein modification, ion exchanges and nucleic acid conformational changes that might cause positive, adaptive or destructive effects and the modulation of EMF can determine the benefit or the severity of the outcomes”* (the study referred to effects coming from radio frequencies close to 900 MHz) [40]. Investigations on detrimental effects coming from external EMFs range from reactions of unicellular organisms (low growth, crippled shape when near to a GSM-telephone using a frequency of 900 MHz) [41] to complex phenomena in multicellular organisms such as behavior and cognition reviewed for effects of microwave exposure [42]. The sensitivity of life goes beyond EMFs, as, e.g., strong evidence exists regarding the effects on the incidence of cancer coming from cosmic ray modulation [43], geomagnetic activities or sun dynamics on (patho)physiology [44,45], or presumed corresponding contributions from geomagnetic reversals [46].

Examples of direct effects of electric and magnetic fields (of extremely low frequency range, e.g., 60 Hz) on charge transfer, and structural changes of proteins or DNA driven by such changes are reviewed [47] (Figure 1a).

## 4. The Significance of the Electromagnetic Fields of the Cell for Life Processes

Cells produce EMFs and apparently cannot avoid doing so. Consequently, EMFs are assumed to not only adjust to cellular changes, e.g., due to variable incoming signals being chemical or physical. They can also feedback on the material (chemical) aspect and signals of the cell. This is supported, e.g., by experiments with glass or quartz barriers delivering strong evidence on the induction of typical cellular processes such as mitosis [17], energy uptake [18] or protein synthesis [14]. While these examples refer to electromagnetic waves, convincing studies also report of membrane voltage alterations leading to stem cell differentiation, gene activation or epigenetic changes [31]. In either case, we learn about influences of non-molecular origin having effects on molecular aspects of cells. Electromagnetic fields external to the organism have measurable effects on life, too, and therefore belong to the environment of cells and organisms [39,40,48]. Whether all effects coming from external fields of the non-biological or the biological environment act via photoreceptors or via resonance from external onto internal fields still needs our shared attention and therefore research. A first conclusion that we can make here is that we cannot understand life without learning more about its internal EMFs. This may also regard evolution.

Second, how much of an organism is due to physical laws ruling life regarding cell-internal EMFs? In some cases of barrier-experiments with the Ciliate *Paramecium caudatum*, the cell populations exposed to one another in proximity but separated by a glass or quartz wall were kept in *total darkness* for 48 hours [18]. Such total absence of external visible light—even at night—might only rarely or not at all be found under natural circumstances of the fresh-water living Ciliate *P. caudatum*. Therefore, results obtained from such experiments may not necessarily be understood as adaptation due to natural circumstances and, hence, selection [49]. Such results are, e.g., the adaption of energy uptake or cell division induced across glass and/or quartz separators in a tester population [18], and further, evidence of cell density being regulated electromagnetically has been reported [19]. We might deduce for these cases that the effects, therefore, belong to the world of physics acting on life. Remembering that charged molecules produce fields in and outside of cells, we may expect and conclude that electromagnetic fields and cells (as constructs of charged biomolecules) produce complex and dynamic reciprocal causalities. Life, in the end and to no surprise, must obey the purely physical laws of electrostatics and electrodynamics and seems to also interact with these laws [50] by using them for communication means beyond purely chemical signaling, and also electromagnetically, besides mechanically or acoustically.

While we realize how much of an organism’s traits are due, e.g., to predation, herbivory, mating and other typical behavioral or physiological adaptions, we do not understand much about the physics of electromagnetism contributing to the traits of organisms. The effects of electromagnetic physics in biological systems await continued discoveries. We have not yet studied the internal cellular EMFs on a broad scale.

Third, evolution is supposed to work through heritable elements only. For a long time, this was thought of as allele changes in chromosomal, nuclear DNA. Recently, extra-chromosomal circular DNA was discovered [51]. Apparently, these non-genomic genetic elements are inherited maternally and, when present, seem to play a role in the development of cancer. What if cellular qualities not transmitted via nucleic acids at all are being inherited from generation to generation, maybe in the form of electromagnetic fields providing a certain pattern of information for development and characteristics of an organism? To be clear, these fields are assumed to be inherited, e.g., via the structure of the zygote and they are in turn influencing gene expression and regulation of cell dynamics in general (Figure 1c). But it remains an open question whether altered fields would lead to stable or repeatable forms of information influencing evolution at population or even higher taxonomic level(s).

The proposed double-aspect of life implies that we must look at both the material components and the EMFs they generate. Together, these two aspects display the dynamics of life. Looking at only one aspect might lead to incomplete views in both basic and applied research. We have the chance to better understand life when we see its double-aspect of field and matter described as a unit that is assumed to co-evolve.

We have learnt much about life and its internal processes, and this goes on, e.g., with regard to the theory of its evolution [52,53,54]. The present paper suggests an integration of electromagnetic fields into research and their implications on living organisms into the theory of life.

## 5. Conclusions

Cell-internal EMFs exist to a much larger extent than has been described in text-books so far. Enough evidence has been accumulated to motivate studying more of their functionality. Yet, we cannot understand them only by applying what we understand so far from a purely molecule-based interpretation of life as EMFs are non-local, weightless, and transmit at much higher speed than diffusing molecules. But cell-matter and cell-EMFs are non-separable, and they are suggested to be understood as a unit that co-evolves, referred here as the double-aspect of life.
